# Anethole Isomerization and Dimerization Induced by Acid Sites or UV Irradiation 

**DOI:** 10.3390/molecules15075012

**Published:** 2010-07-22

**Authors:** Hans T. Castro, Jairo René Martínez, Elena Stashenko

**Affiliations:** Chromatography Laboratory, CIBIMOL, Research Center of Excellence CENIVAM, Building 45, Universidad Industrial de Santander, Carrera 27 calle 9, Bucaramanga, Colombia

**Keywords:** *trans*-anethole, photoisomerization, HY zeolite, *cis*-anethole, dimerization

## Abstract

The formation of *cis*-anethole and various dimers as a result of the exposure of *trans*-anethole to microporous solid acids (dealuminated HY zeolites), or UV-Vis irradiation was established by means of high resolution gas chromatography coupled to mass spectrometry. 3,4-bis-(4-Methoxyphenyl)-(*E*)-hex-2-ene was the most abundant compound among eight different methoxyphenyl-disubstituted hexenes produced by electrophilic addition and elimination reactions induced by HY zeolites. (1a,2a,3b,4b)-1,2-bis(4-Methoxyphenyl)-3,4-dimethylcyclobutane was the principal component in the mixture of 5 methoxyphenyl-disubstituted cyclobutanes found, together with *cis*-anethole, after UV-Vis irradiation of a *trans*-anethole solution in toluene.

## 1. Introduction

*trans*-Anethole (**1a**) is one of the main constituents of anise, clove, cinnamon and thyme essential oils [1,2,3]. *trans*-Anethole-containing oils are widely used in the food and liquor industries [4], although their use has been the subject of discussion due to the formation of *cis*-anethole (**1b**) when *trans*-anethole is exposed to UV radiation or acidic conditions. *cis*-Anethole is toxic, and possesses an unpleasant scent and flavor [5]. We have previously reported that isomerization of *trans*-anethole takes place during the catalytic transformation of anise oil over zeolite Y [6]. 

Zeolites are porous crystalline aluminosilicates formed by [SiO_4_]^-4 ^and [AlO_4_]^-5^ tetrahedral units. The relative amounts of these two building blocks determine the negative charge of the framework. This charge is compensated by the presence of counterions such as sodium, calcium or proton. Protonic dealuminated zeolites have found important applications as petroleum cracking catalysts. They contain Brønsted-type acid sites associated to framework Al [7]. Their use as catalysts is mainly related to their high acidity [8]. In this work we present the results of GC-MS analysis of the products (which included anethole dimers) resulting from the exposure of *trans*-anethole to HY zeolites, at 30, 60 and 90 °C. Details of the hydrogen transfer between zeolite and *trans*-anethole were based on GC-MS analysis of the reaction products obtained when deuterated acid zeolites, HDY, were used.

*trans*-Anethole exposure to UV radiation and its chemical transformation have been investigated by different authors. Lewis and Kojima [9,10] studied *trans*-anethole photoisomerization at two excitation wavelengths, λ_exc_ (281 and 313 nm), under different reactant concentrations, and in the absence or presence of various electron acceptors. Among their results, the authors highlighted the formation of *cis*-anethole and five dimers, products of [2+2] cycloaddition and electron transfer. One dimer (**6e** in our work) was reported as the only one formed from *trans*-anethole; whereas a different dimer (**6c** in our work) was formed from *trans*-anethole only in the presence of electronic acceptors (cyanoanthracene, CA, 9,10-dicyanoanthracene, DCA, and 1,4-dicyanobenzene, DCB). We present results on the study of UV-Vis-induced isomerization and dimerization of *trans*-anethole in toluene, which indicate that dimer formation could take place without the addition of electronic acceptors. 

## 2. Results and Discussion

### 2.1. trans-Anethole treatment with HY zeolites

**Scheme 1:**
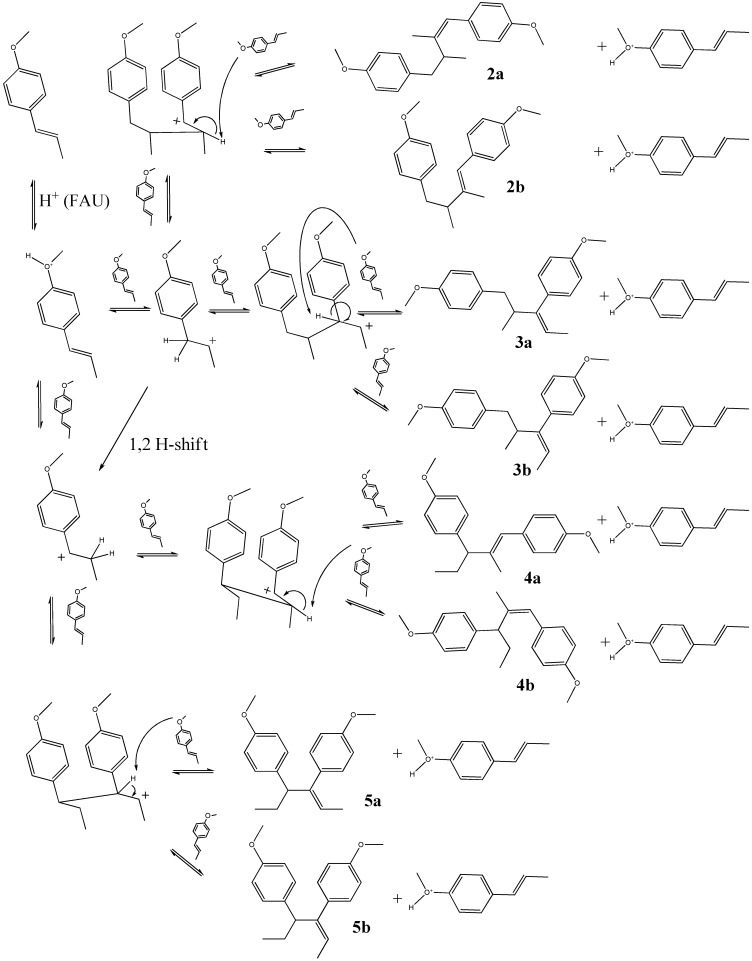
Proposed formation routes for dimers **2a - 5b** from *trans*-anethole (**1a**).

A total of nine compounds (> 0.1%) were tentatively identified by means of GC-MS of the mixture obtained after exposure of *trans*-anethole to HY zeolites at 30, 60 and 90 ºC, for 5 h. The distinction between geometrical isomers was based on the comparison of their molecular ion abundances in their mass spectra, and some characteristic ion-fragments. (*E*)-geometry was associated with the more stable molecular ion of the two isomers. Since there were no standard substances available, dimer structure assignment relied strongly on the comparison of typical fragment losses found in their mass spectra and on the structures which appear in Scheme 1. Table 1 presents the relative amounts (%) of *trans*-anethole and its transformation products. The structural formulae of dimers **2a–5b** appear in Scheme 1. Dimer **5b** (20–63%) was the most abundant compound obtained under the conditions presented in Table 1. *cis*-Anethole (**1b**, 1–2%), the dimers [M^+•^, m/z 296] **2a **(0.4–2.7%), **2b **(0.7–4%), dimers **3a** and **3b **(1–23%), **4a** (0.4–5%), **4b** (0.71–11%) and **5a **(<0.1%) were also found as transformation products.

**Table 1:** Relative amounts of *trans*-anethole and its transformation products (>0.1%), after its exposure to HY zeolites (5 h).

		Relative amount, %^a^								
	Zeolite	HY1			HY2			HY3		
Compd.	T, °C	30	60	90	30	60	90	30	60	90
c *i*s-Anethole		0.3 ± 0.02	1.9 ± 0.24	0.8 ± 0.01	0.2 ± 0.01	1.2 ± 0.80	0.9 ± 0.11	0.2 ± 0.03	1.2 ± 0.08	0.4 ± 0.06
*trans*-Anethole		99 ± 0.02	45.0 ± 0.80	9.0 ± 0.86	99 ± 0.01	30 ± 23	12 ± 1.1	99.0 ± 1.7	76 ± 1.4	8.0 ± 1.9
**2a**		---	2 ± 0.17	3 ± 0.16	---	2.7 ± 0.92	2 ± 0.08	---	0.4 ± 0.18	1.4 ± 0.17
**2b**		---	1.0 ± 0.08	0.9 ± 0.03	---	1.7 ± 0.74	4 ± 0.24	---	0.7 ± 0.05	2.6 ± 0.27
**3a.** **3b**		---	2.0 ± 0.06	9.0 ± 0.03	---	5 ± 2.02	9 ± 0.09	---	1.0 ± 0.19	23 ± 2.2
**4a**		---	1.0 ± 0.08	3 ± 0.04	---	1.0 ± 0.38	5 ± 0.06	---	0.45 ± 0.05	2.2 ± 0.18
**4b**		---	2.0 ± 0.05	11 ± 0.11	---	3.0 ± 1.14	7 ± 1.3	---	0.71 ± 0.06	5.9 ± 1.2
**5a**		---	*tr*	*tr*	*---*	*tr*	*tr*	*---*	*tr*	*tr*
**5b**		---	45 ± 0.30	63 ± 0.92	---	55 ± 18	60 ± 0.4	---	20 ± 1.2	57 ± 5.6

^a^ Mean ± s (n = 3); *tr =* trace (<0.1%)

The relative amount of dimers increased with temperature (Table 1), whereas *cis*-anethole was formed in higher amount at 60 °C than at either 30 or 90 °C. This suggests that competition between isomerization and dimerization exists and it is consistent with the existence of a common intermediate, *i.e*. protonated anethole, for both processes. The increase of the dimers as the temperature was incremented could be explained by a higher reaction rate for dimerization than for isomerization. 

There are very few publications in the scientific literature on the dimers presented in Table 1. Structures **3a**–**5b** appeared in studies on biological activity and synthetic estrogens carried out in the first half of the last century [11,12]. Dimer **5b** was mentioned by Whitmore in the context of a study on olefin polymerization with acid catalysts [13]. Scheme 1 contains the proposed formation scheme for dimers **2a**–**5b**. The two intermediary carbocations resulting from protonation of the *trans*-anethole double bond bind each one with a neutral molecule at two different locations. The most stable carbocation, stabilized by resonance, leads to dimers **4a**–**5b**. The second carbocation produces the less abundant dimers **2a**–**3b**. The orientation of the attack of the incoming *trans*-anethole molecule on the carbocation determines whether the **a** or **b** isomer is formed. Although only the *trans*-isomer was employed in the scheme proposed in Scheme 1, in principle, some of the dimers **2a**–**5b** could also result from the addition of a molecule of *cis*-anethole to either cationic intermediary. However, the very low *cis*-anethole amounts make this a rare event.

### 2.2. Transformation of trans-anethole exposed to deuterated zeolite, HDY

**Figure 1:**
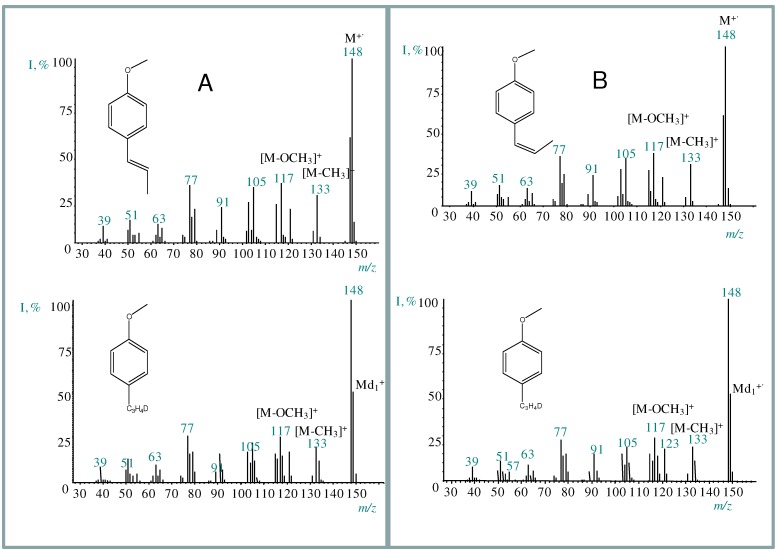
Mass spectra (EI, 70eV) of anethole isomers and their deuterated analogs formed after the exposure of *trans*-anethole to deuterated acid faujasite, HDY. **A**. *trans*-Anethole and its isotopomer. **B**. *cis*-Anethole and its isotopomer.

Based on the *in situ *^13^C-NMR determination of alkoxides, various studies [14,15,16,17,18,19,20] have proposed a carbenium-mediated mechanism for *trans*-anethole isomerization or oligomerization. Since both reactions take place in the same medium, it is possible that some dimers result from the reaction of a just-formed *cis*-anethole molecule with the cation obtained from the highly abundant *trans*-anethole. As evidenced in their mass spectra (Figure 1), peaks corresponding to *cis*- or *trans*-anethole fragmentation products which do not contain methoxy groups, incorporate deuterium. The presence of deuterium in both *cis*- and *trans*- anethole after the treatment of pure *trans*-anethole with deuterated faujasite supports the proposed formation scheme in which isomer interconversion occurs (Scheme 1). 

The *cis*-anethole mass spectra of Figure 1 were obtained from the GC-MS analysis of the products of *trans*-anethole exposure to either acidic faujasite, HY, or deuterated acidic faujasite, HDY, allowing us to affirm that *cis*-anethole formation was due to the zeolite treatment. The ratio of characteristic fragment ion abundances in the mass spectrum of deuterated *cis*-anethole (*m/z* 117/118 = 22/26; *m/z* 133/134 = 19/18; *m/z* 148/149 = 100/77), compared to those of *cis*-anethole formed on the non-deuterated faujasite HY (*m/z* 117/118 = 33/5; *m/z* 133/134 = 25/4; *m/z* 148/149 = 100/14), showed an increased abundance of deuterated *cis*-anethole when HDY was used. Thus, an electrophilic deuterium addition to the sp^2^ carbon of the *trans*-anethole double bond, takes place during the isomerization or dimerization processes. 

**Scheme 2:**
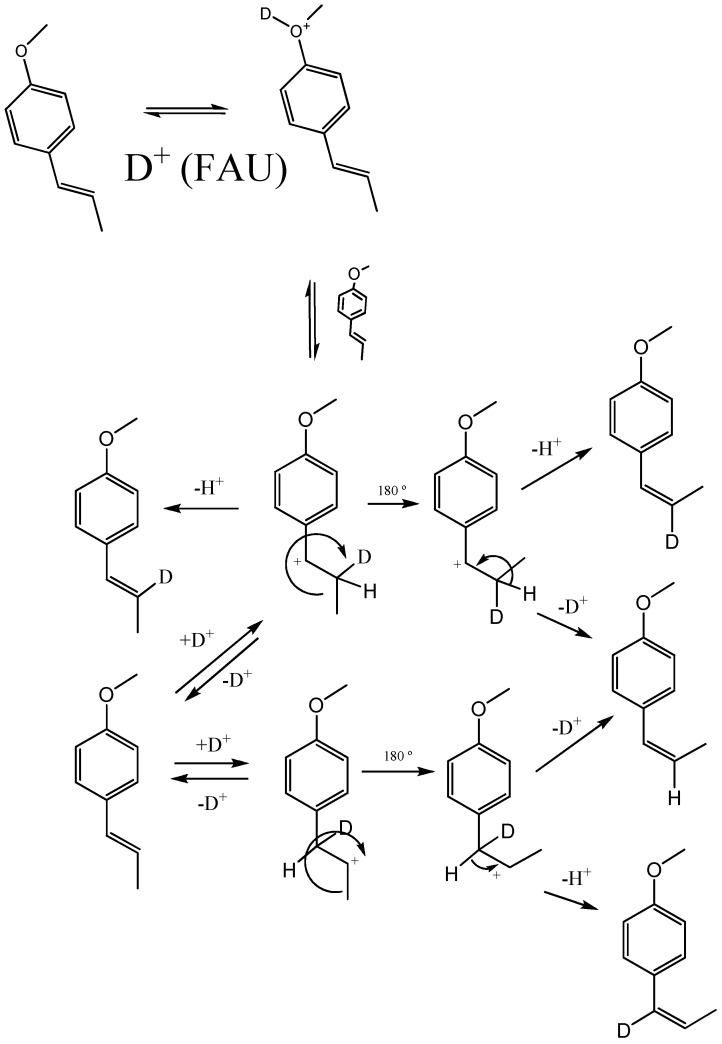
Proposed scheme for *trans*-anetholeisomerization over deuteratedfaujasite, HDY.

The ratios of the ion abundances at *m/z* 148 and 149 of deuterated *trans*-anethole are similar to those observed in the deuterated *cis*-anethole mass spectrum. This agrees with the existence of an intermediary carbocation that gives rise to either isomer at a similar rate. An isomerization scheme is proposed in Scheme 2. The relatively high abundance of *m/z* 149 in the mass spectra of isomerization products (both *cis*- and *trans*-antehole), indicative of the presence of deuterium, suggests that the neutral product may be formed by the loss of a proton different from that which participated in the electrophilic addition.

**Figure 2:**
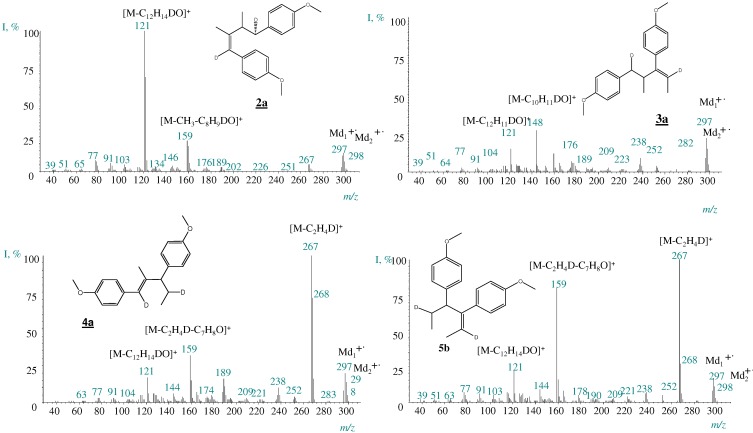
Mass spectra (EI, 70 eV) of anetholedimers formed as a result of *trans*-anethole exposure to deuteratedfaujasite HDY.

**Scheme 3:**
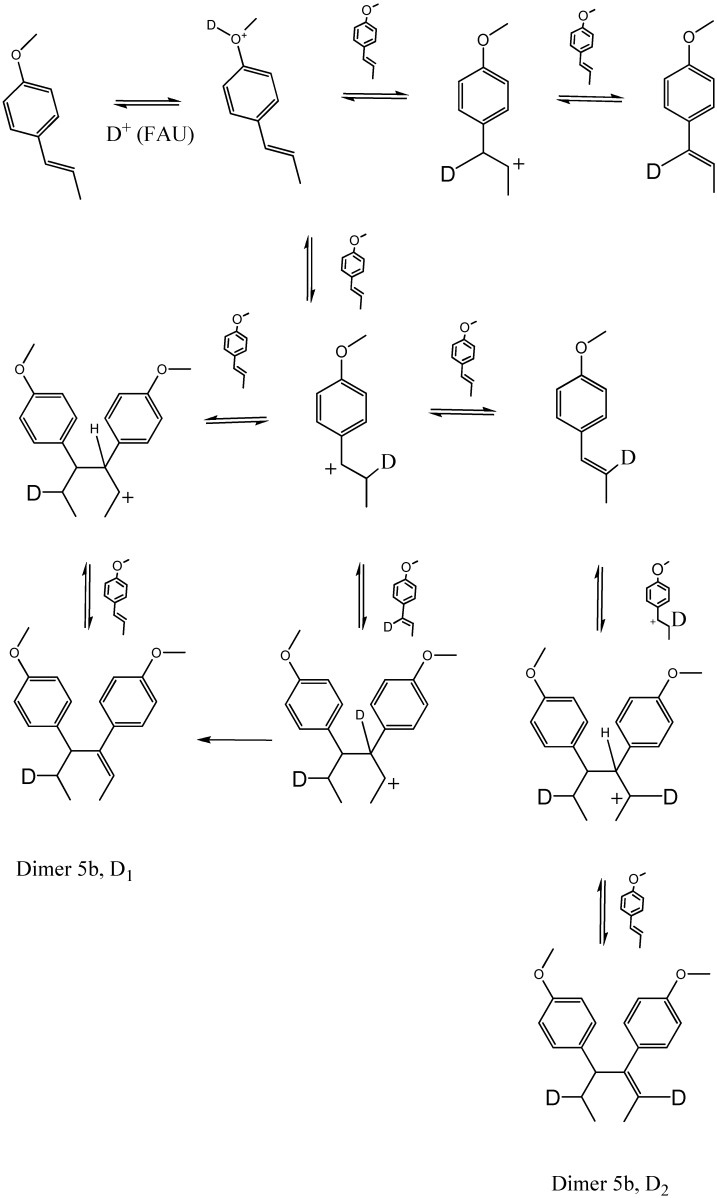
Proposed mechanism for the formation of singly and doubly deuterateddimers **5b** obtained after the exposure of *trans*-anethole to deuteratedfaujasite, HDY.

Typical mass spectra of the dimers formed after *trans*-anethole exposure to deuterated faujasite HDY, appear in Figure 2. Ions at *m/z* 297 and 298 in the mass spectra of all dimers indicate single and double deuteration, respectively, which results from the participation of one or two deuterated molecules of either *cis*- or *trans*-anethole. The signal of the non-deuterated molecular ions (*m/z* 296) was in all cases of lower intensity than that of the monodeuterated dimers (*m/z* 297). Scheme 3 shows a mechanism proposed for the dimerization process, applied to the case of dimer **5b**, which was the most abundant product. In the observed mass spectra (electron impact ionization, 70 eV), peaks of the various deuterated ions derived from these structures were of high intensities (Figure 2).

### 2.3. trans-Anethole phototransformation

**Figure 3:**
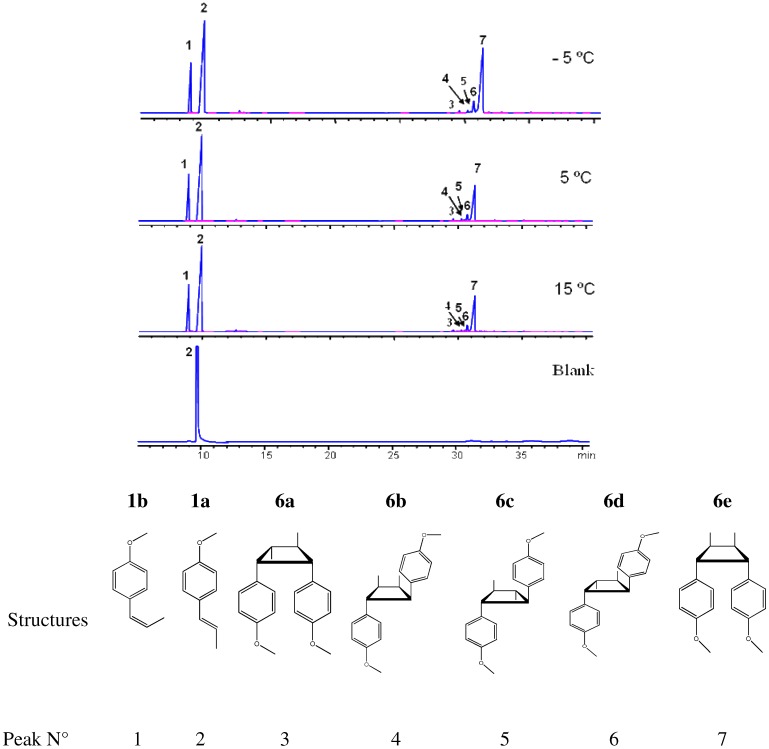
Chromatographic profiles of the different products obtained during *trans*-anethole photoreaction (UV-Vis) in toluene at –5, 5, and 15 °C for 2 h. 1. *cis*-Anethole; 2. *trans*-anethole; 3. Dimer **6a**; 4. Dimer **6b**; 5. Dimer **6c**; 6. Dimer **6d**; 7. Dimer **6e**.

Figure 3 shows the chromatographic profiles of the mixtures that resulted from the irradiation of a *trans*-anethole toluene solution with UV-Vis at –5, 5 and 15 °C for 2 h. In addition to *cis*-anethole, five other compounds were identified at relative amounts above 0.1% in all cases. Anethole (*cis-* and *trans‑*) and their dimers were tentatively identified according to reported data [9,10] and the comparison of their mass spectra (EI, 70 eV) fragmentation patterns, and calculated dipole moments and ionization potentials (Table 2). The interaction of an S_1_ excited *t-A* molecule with another one in its ground state, S_o_ in a [2 + 2] cycloaddition leads to dimers **6c** (anti head-to-head) and **6e** (syn head-to-head). The structure of dimer **6e**, the most abundant product (peak 7) in the chromatograms (Figure 3), was assigned by Nozaki *et al*. [21] based on independent synthesis and NMR spectra. This dimer was the only sensitizer-free *trans*-anethole dimerization (313 nm) product reported by Lewis and Kojima [9,10]. The mass spectrum of dimer **6c** had been reported by Meyer and Metzger [22]. Dimer **6c **(peak 5), the second most-abundant product in the chromatograms (Figure 3), was obtained in Lewis and Kojima’s experiments when a sensitizer (1,4-dicyanobenzene, cyanoanthracene) was present. Marquez *et al*. included dimer **6c** as one of the products of *trans*-anethole electron transfer reaction with radical cation tri-(4-bromophenyl) ammonium [23]. 

**Table 2:** Anetholedimers **6a**–**6e** formed during *trans*-anethole photoreaction.

Dimer	Molecular structure, R = *p*-Methoxy-phenyl	DB-5 column		t_R_, min (Polar column, DB-WAX)	Dipole moment, Debye	EI (eV)
		t_R_, min (Fig. 3)	RI			
**6a**	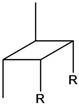	29.6	2139	---	2.11	7.90
**6b**	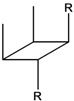	30.2	2106	---	3.00	8.30
**6c**	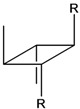	30.9	2266	142.8	2.54	8.10
**6d**	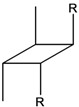	30.5	2289	---	2.53	8.29
**6e**	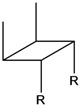	31.1	2255	151.4	2.37	8.27

**Figure 4:**
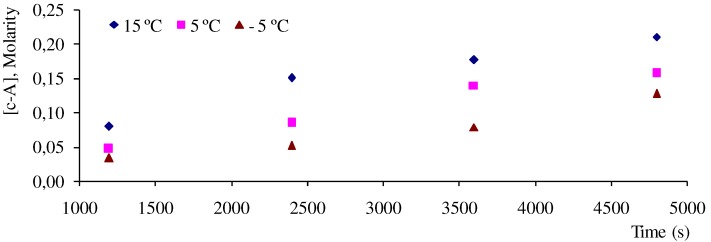
Variation of *cis*-anethole concentration during *trans*-anethole UV-Vis iiradiation.

**Scheme 4:**
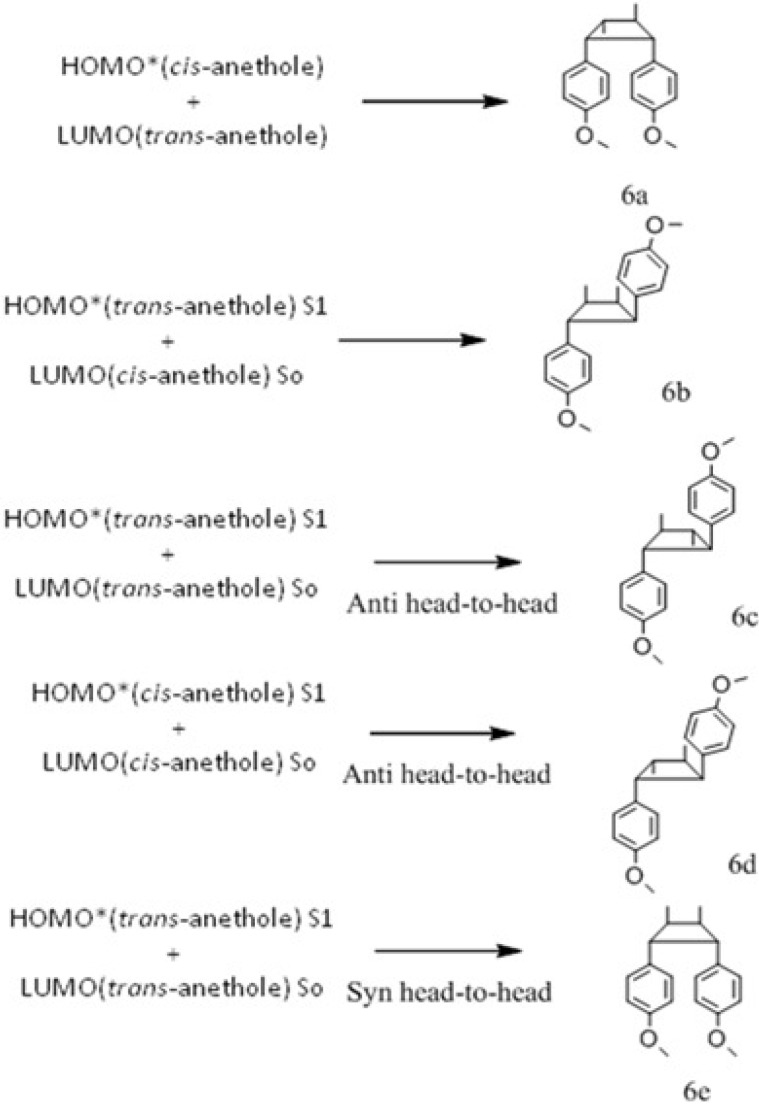
Scheme proposed for *trans*-anetholephotodimerization products.

Dimers **6a**, **6b** and **6d** have been found among *cis*-anethole photodimerization (at 313 nm) products, but not among *trans*-anethole irradiation products, formed in the presence or absence of sensitizer [10,24]. Their formation during *trans*-anethole UV-Vis irradiation may be explained by the reaction of *cis*-anethole formed during the experiment. Figure 4 shows a continuous increase of *cis*-anethole concentration during the experiment (2 h) at the three temperatures employed. The relative amounts of **6a**, **6b** and **6d** dimers showed a small increase. Thus, we conclude that the rate of *trans*-anethole photoisomerization is higher than that of *cis*-anethole photodimerization. Dimer **6d** formation was linked in Lewis and Kojima’s work with oxygen sensitization of *trans*-anethole. In the present work, *trans*-anethole UV-Vis irradiation experiments were conducted in a sealed reactor that had been maintained under dry nitrogen flow to remove molecular oxygen. No epoxides were found among the UV-Vis irradiation products. The scheme of formation proposed for the various photodimerization products is shown in Scheme 4.

Caldwell *et al.* [24] observed *cis*-anethole configuration retention in [2+2] reactions with singlet 9-cyanophenanthrene. The dimer structures proposed here maintain the *cis*-anethole stereochemistry. Dimerizations result from ^1^*t*-A*-^1^*t*-A, and to a lesser extent, from ^1^*t‑*A*-^1^*c*-A interactions. The latter happens with lower frequency due to the low *cis*-anethole concentration. Table 3 presents conversions and selectivities determined after UV-Vis *trans*-anethole toluene solution irradiation for 2 h at -5, 5 and 15 °C. *cis*-Anethole and dimer **6e** were the most abundant products. While *trans*-anethole conversion increased with temperature, the selectivity towards *cis*-anethole decreased, whereas those of **6c** and **6e** dimers increased. This shows the competition that exists between isomerization and dimerization.

**Table 3:** Conversion and selectivities of *trans*-anethole (in toluene) photoreaction products (UV-Vis, 120 min, -5, 5 and 15 °C).

T ºC	*trans*- Anethole conversion, %	Selectivity, %					
		*cis*-anethole	6a	6b	6c	6d	6e
-5	30	42	<1	2	5	1	50
5	35	38	<1	2	5	2	54
15	52	34	<1	<1	8	1	57

### 2.4. Quantum yield of trans-anethole photoisomerization

**Figure 5:**
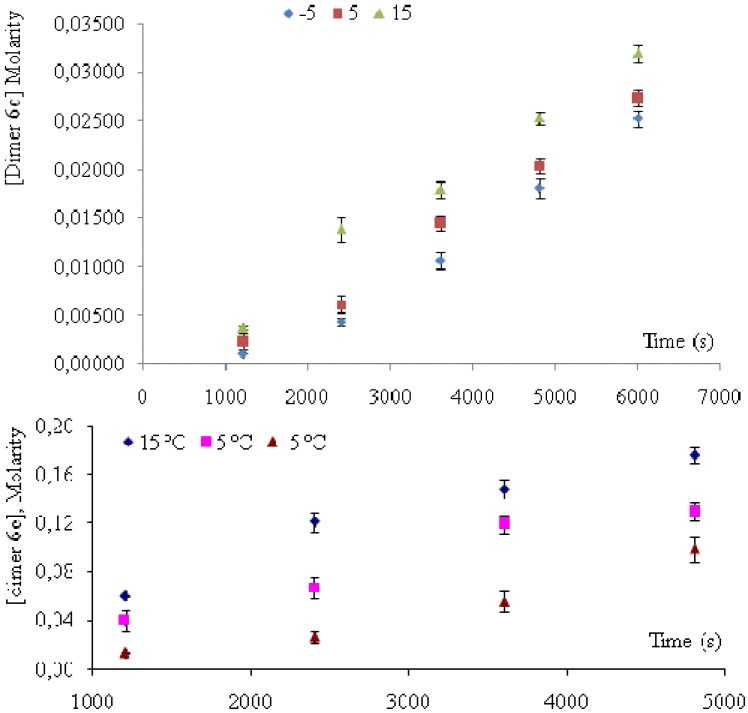
Concentration profiles of dimers **6c** and **6e** during *trans*-anethole UV-Vis irradiation for 2 h.

Quantum yields of the formation of *cis*-anethole (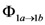) and **6c** (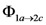) and **6e** (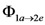) dimers at ‑5, 5 and 15 °C, were calculated as the ratio of reaction rate over flux of absorbed photons (Table 4). In each case, the reaction rate was calculated as the slope of the plot of concentration Vs time. Figure 4 and Figure 5 show the linear nature of the concentration profiles of *cis*-anethole and dimers **6c** and **6e**. Potassium ferrioxalate solutions were employed in the actinometric determination of flux as 3 × 10^‑4^ ± 2 × 10^-5^ mol photon L^-1^ s^-1^. 

**Table 4:** Quantum yields determined for *trans*-anetholeisomerization and dimerization.

T (ºC)	*cis*-Anethole ^a^ 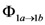	6c Dimer ^b^ 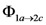 × 10^ 6^	6e Dimer ^c^ 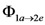
-5	0.09	6	0.08
+5	0.11	5	0.09
+15	0.12	5	0.10

**^a^** CV(%) = 1.1%; **^b^** CV(%) = 1.6%; **^c^** CV(%) = 1.2%; n = 3

The monotonous increase of *trans*-anethole photoisomerization and dimerization products (Figure 4 and Figure 5) is consistent with a higher rate for the reaction of excited singlet *trans*-anethole (^1^*t*-A*) with *trans*-anethole than that observed for its reaction with *cis*-anethole. Since *cis*-anethole is being formed, encounters of ^1^*t*-A* with *cis*-anethole are much less likely than those with *trans*-anethole. Additionally, *cis*-anethole has been reported as less reactive than *trans*-anethole in the photocycloaddition with 9-cyanophenanthrene and 6-methylphenantridine [24]. The much higher abundance of **6c** and **6e** dimers in comparison with **6a** and **6b** dimers is another consequence of this reaction rate difference. 

The quantum yields obtained (Table 4) are close to those reported for *trans*-anethole photoisomerization in hexane and acetonitrile (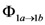 = 0.12 and 0.13, respectively) [26]. The increase of quantum yields with temperature indicates the existence of an energy barrier in photoisomerization and photodimerization. The 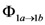 values determined after multichromatic UV-Vis irradiation of *trans*-anethole in toluene (0.35 M) are similar to the values reported for *trans*-anethole photoisomerization after monochromatic (254, 281, or 313 nm) irradiation [9,10]. Thus, there is no appreciable dependence of quantum yield on excitation wavelength. The quantum yield for *trans*-retinal photoisomerization (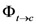
~ 0.67) has been found to be independent of excitation wavelength [25]. On the other hand, conformational photoisomerization [26] of *trans*-stilbene and stilbene derivatives [27], some fluorinated compounds [28], and azobenzene and its dimers [29,30,31,32,33,34] has been reported as irradiation wavelength dependent.

## 3. Experimental

### 3.1. General

*trans*-Anethole (99%), ammonium nitrate (98%) D_2_O (99.96% deuteration), NaY zeolite, were obtained from Aldrich Chemical Co. Inc (Milwaukee, WI, USA). Toluene (99%, reagent grade) and DCl (20% in D_2_O, 99.5% deuteration) were purchased from Merck (Darmstadt, Germany). *cis-*Anethole chromatographic reference data were obtained from hydrodistilled anise seed essential oil. HCl (ACS grade, 37%) was obtained from J. T. Baker (Phillipsburg, NJ, USA). 

Chromatographic quantification was performed with an HP 5890A Series II (Hewlett-Packard, Palo Alto, CA, USA) gas chromatograph equipped with a split/splitless injection system (split ratio 6:1), a flame ionization detector (FID) and a DB-5 (J&W Scientific, Folsom, CA, USA) capillary column (30 m × 0.32 mm × 0.25 μm) coated with 5%-phenyl-poly(methylsiloxane). Synthetic air (zero grade), H_2 _(99.995%), N_2_ (99.995%), and He (99.995%), were obtained from Aga-Fano S.A. (Bucaramanga, Colombia). The oven temperature was programmed from 80 ºC (1 min) @10 ºC/min to 230 ºC (5 min). The injection port and detector temperatures were maintained at 250 and 280 ºC, respectively. Chromatographic data were processed with HP ChemStation A.06.03 (Hewlett-Packard*,* Palo Alto, CA, USA) software.

Two GC–MS systems were employed: an Agilent Technologies 6890 Plus gas chromatograph (Palo Alto, CA) equipped with an Agilent Technologies 5973N mass selective detector (EI, 70 eV, *m/z* 40–350) and an Agilent Technologies 6890 gas chromatograph coupled to an Agilent Technologies 5975 mass selective detector (EI, 70 eV, *m/z* 40–350). Both systems were equipped with a split/splitless injector (split ratio 1:30), a 7863 automatic injector and an MS-ChemStation G1701-DA data system that included the spectral libraries WILEY 138K, NIST 2002, and QUADLIB 2004. A fused-silica 5% phenylpoly(dimethylsiloxane) capillary column (DB-5MS, J&W Scientific) of 60 m, 0.25 mm i.d., 0.25 μm, d_f_, and a fused-silica cross-linked, bonded poly(ethylene glycol) capillary column (DB-WAX, J & W Scientific, Folsom, CA, USA) of 60 m, 0.25 mm, i.d. 0.25 μm, d_f_, were employed. The oven temperature program employed with the DB-5MS column was from 100 °C (3 min) to 200 °C (5 min) at 10 °C/min, then to 280 °C (40 min) at 15 ºC/min. For the DB-WAX column, the oven temperature was programmed from 100 °C (3 min) to 200 °C (5 min) at 10 °C/min, then to 220 °C (40 min) at 4 °C/min. The ionization chamber and the transfer line temperatures were kept at 230 °C and 285 °C, respectively. The following are the mass spectral data of the various dimers encountered as *trans*-anethole photoreaction products.

*(Z)-1,4-bis(4-Methoxyphenyl)-2,3-dimethylbut-1-ene* (**2ª**). W_M+·_ (4.8%). *m/z* (I%): M^+^· 296(12), [M-C_10_H_12_O]^+ ^267(7), [M-C_2_H_5_-C_7_H_8_O]^+^ 159(28), [M-C_12_H_15_O]^+^ 121(100). 

*(E)-1,4-bis(4-methoxyphenyl)-2,3-dimethylbut-1-ene* (**2^b^**). W_M+·_ (5.8%). *m/z* (I%): M^+^· 296(13), [M-C_2_H_5_]^+ ^ 267(5), [M-C_2_H_5_-C_7_H_8_O]^+ ^159(24), [M-C_10_H_12_O]^+ ^148(1), [M-C_12_H_15_O]^+ ^121(100).

*(Z)-3,5-bis(4-methoxyphenyl)-4-methylpent-2-ene* (**3^a^**). W_M+·_ (1.7%). *m/z* (I%): M^+^·296(4), [M-C_2_H_5_-C_7_H_8_O]^+ ^159(2), [M-C_10_H_12_O]^+ ^148(100), [M-C_12_H_15_O]^+ ^121(38).

*(E)-3,5-bis(4-methoxyphenyl)-4-methylpent-2-ene* (**3^b^**). W_M+·_ (1.3%). *m/z* (I%): M^+^· 296(3), [M-C_2_H_5_-C_7_H_8_O]^+ ^159(3), [M-C_10_H_12_O]^+ ^148(100), [M-C_12_H_15_O]^+ ^121(34).

*(E)-1,3-bis(4-methoxyphenyl)-2-methylpent-1-ene* (**4^a^**). W_M+·_ (7.6%). *m/z* (I%): M^+^· 296(28), [M-C_2_H_5_]^+ ^267(100), [M-C_2_H_5_-C_7_H_8_O]^+ ^159(28), [M-C_10_H_12_O]^+ ^148(2), [M-C_12_H_15_O]^+ ^121(17).

*(Z)-1,3-bis(4-methoxyphenyl)-2-methylpent-1-ene* (**4^b^**). W_M+·_ (5.0%). *m/z* (I%): M^+^· 296(24), [M-C_2_H_5_]^+ ^267(100), [M-C_2_H_5_-C_7_H_8_O]^+ ^159(31), [M-C_10_H_12_O]^+ ^148(1), [M-C_12_H_15_O]^+ ^121(19).

*(Z)-3,4-bis(4-methoxyphenyl)hex-2-ene* (**5^a^**). W_M+·_ (6.5%). *m/z* (I%): M^+^· 296(34), [M-C_2_H_5_]^+ ^267(100), [M-C_2_H_5_-C_7_H_8_O]^+ ^159(82), [M-C_12_H_15_O]^+ ^121(42).

*(E)-3,4-bis(4-methoxyphenyl)hex-2-ene* (**5^b^**). W_M+·_ (5.2%). *m/z* (I%): M^+^· 296(28), [M-C_2_H_5_]^+ ^267(100), [M-C_2_H_5_-C_7_H_8_O]^+ ^159(98), [M-C_10_H_12_O]^+^ 148(3), [M-C_12_H_15_O]^+ ^121(30).

*(1a,2a,3a,4b)-1,2-bis(4-methoxyphenyl)-3,4-dimethylcyclobutane* (**6^a^**). W_M+·_ (6.6%). *m/z* (I%): M^+^· 296(4), [M-CH_3_]^+ ^267(1), [M-C_4_H_8_-H]^+^ 249(1), [M-CH_3_-C_7_H_8_O]^+^ 175(4), [M-C_10_H_12_O]^+^· 148(100), [M-C_12_H_15_O]^+ ^121(12). 

*(1a,2b,3a,4a)-1,2-bis(4-methoxyphenyl)-3,4-dimethylcyclobutane* (**6^b^**). W_M+· _(4.7%). *m/z* (I%): M^+^· 296(81), [M-CH_3_]^+ ^267(9), [M-C_4_H_8_-H]^+^ 249(15), [M-CH_3_-C_7_H_8_O]^+ ^175(36), [M-C_10_H_12_O]^+^· 148(67), [M-C_12_H_15_O]^+ ^121(100).

*(1a,2b,3a,4b)-1,2-bis(4-methoxyphenyl)-3,4-dimethylcyclobutane* (**6^c^**). W_M+· _(0.6%). *m/z* (I%): M^+^· 296(<1), [M-C_10_H_12_O]^+^· 148(100), [M-C_12_H_15_O]^+ ^121(4).

*(1a,2b,3b,4a)-1,2-bis(4-methoxyphenyl)-3,4-dimethylcyclobutane* (**6^d^**). W_M+· _(1.2%). *m/z* (I%): M^+^· 296(88), [M-CH_3_]^+ ^267(8), [M-C_4_H_8_-H]^+^ 249(9), [M-CH_3_-C_7_H_8_O]^+ ^175(38), [M-C_10_H_12_O]^+^· 148(34), [M-C_12_H_15_O]^+ ^121(100).

*(1a,2a,3b,4b)-1,2-bis(4-methoxyphenyl)-3,4-dimethylcyclobutane* (**6^e^**). W_M+· _(0.5%). *m/z* (I%): M^+^· 296(<1), [M-C_10_H_12_O]^+^· 148(100), [M-C_12_H_15_O]^+ ^121(4).

### 3.2. Faujasite preparation and characterization

NaY zeolite (10 g) was suspended at 60 ºC for 2 h in an aqueous ammonium nitrate solution (1M, 150 mL) under continuous stirring (1,000 rpm). It was dried afterwards (100 °C, 12 h) and placed in a quartz tube (1 cm I.D.) in a cylindrical oven and heated to 550 °C under a steam flow at 1.38 bar. Heating periods of 2, 4 and 6 h led to the formation of zeolite samples HY1, HY2 and HY3, respectively. They were subsequently treated with HCl (0.5 M, 6 h, continuous stirring) to remove extra-framework aluminum. 

### 3.3. Zeolite characterization

Dealuminated HY zeolites were characterized by means of elemental analysis (energy dispersive X-ray fluorescence, Shimadzu EDX 800 HS; Columbia, MD, USA), infrared spectroscopy (Bruker, Tensor 27; Billerica, MA, USA) and X-ray diffraction (Rigaku D-MAX-III/B; Tokyo, Japan). 

### 3.4. trans-Anethole treatment with dealuminated HY zeolites

*trans*-Anethole toluene solutions (2 mL, 0,1 M) were placed into three screw-capped test tubes (10 mL), each one containing a different zeolite (zeolites HY1, HY2 and HY3, 25 mg). Zeolites had been previously activated (100 °C, 12 h). Reactions were performed by heating the tubes in a water bath (30, 60 and 90 °C, 5 h). After chromatographic analysis of the resultant mixtures, conversion and selectivities were calculated as follows:

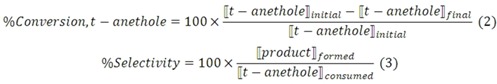



Molecular ion stabilities, W_M+_, were calculated from the dimers’ mass spectra and used to distinguish configuration isomers: according to equation 4, the molecular ion abundance is divided by the sum of abundances of all fragment ions > *m/z* 50:

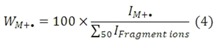



### 3.5. trans-Anethole treatment with deuterated HY zeolite

Zeolite F1 (10 mg) was suspended in DCl/D_2_O (1:9, 1 mL) and heated to 60 °C for 6 h. It was then vacuum filtered and dried (100 °C, 12 h). An infrared spectrum of the resulting material was acquired (Bruker Tensor FT-IR spectrometer). This zeolite sample was then suspended in a test tube (screw cap, 10 mL) with a *trans*-anethole toluene solution (0,1 M, 2 mL) and heated to 60 °C for 5 h under continuous stirring. The tube was filled under dry nitrogen flow. The mixture was decanted and analyzed by GC-MS in selected ion monitoring mode (SIM). The selected ions corresponded to the molecular ion and its main fragments, together with their deuterated analogs (*m/z* 148, 149, 117, 118, 133, 134). 

### 3.6. Phototransformation kinetics

A *trans*-anethole toluene solution (0.35 M, 15 mL) was irradiated in an Ace Glass (Vineland, NJ, USA) 7880 photoreactor assembly with a 5.5 Watt Pen-Ray gaseous Hg discharge UV-Vis lamp, at 5 °C for 120 min under constant stirring (100 rpm). *trans*-Anethole toluene solution aliquots (10 µL) were withdrawn every 20 min and analyzed by GC-FID, using the external standard (*trans*-anethole) method for quantification. 

### 3.7. Quantum yield determination

The Pen Ray UV lamp emission spectra were recorded with a high resolution HRG000c6-UV-NIR spectrometer (Ocean Optics, Dunedin, FL, USA) at the Atomic and Molecular Spectroscopy Laboratory of the Industrial University of Santander (Colombia). Discrete emission lines were found at 255, 313, 366, 405, 437, 548, and 579 nm. Quantum yields were determined as the quotient between the time derivative of the concentration and the flux, according to the following equation, in which X stands for *cis*-anethole, dimer **2c**, or dimer **2e**:

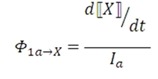
(5)


Potassium ferrioxalate was used for the actinometric determination of Ia, the number of quanta absorbed by *trans*-anethole per unit concentration, per unit time, according to the IUPAC recommended procedure [35]. 

## 4. Conclusions

Isomerization and dimerization are concurrent processes in the *trans*-anethole transformation observed during its exposure to acid sites in a zeolite or UV-Vis irradiation. Proton transfer from the zeolite to *trans*-anethole generates cationic intermediaries which lead the formation of eight different anethole dimers (methoxyphenyl-disubstituted hexenes). Their relative amounts agreed with those expected from the relative stability of the intermediary cationic species. The use of deuterated zeolites showed that the proton eliminated during the isomerization process is not necessarily the same proton transferred to the *trans*-anethole molecule when the cationic intermediary is formed. c*is*-Anethole and five isomeric methoxyphenyl-disubstituted cyclobutanes were observed as products of the transformation induced by the UV-Vis irradiation of *trans*-anethole. 
